# Multicentre comparison of various microaxial pump devices as a bridge to durable assist device implantation

**DOI:** 10.1002/ehf2.15282

**Published:** 2025-04-04

**Authors:** Marta L. Medina, Daniel Lewin, Hendrik Treede, Sebastian V. Rojas, Alexander Bernhardt, Michael Billion, Anna L. Meyer, Ivan Netuka, Janajade Kooij, Marina Pieri, Antonio Loforte, Mauro Rinaldi, Mariusz K. Szymanski, Adriaan O. Kraajieveld, Christian J.H. Moeller, Payam Akhyari, Khalil Jawad, Bastian Schmack, Gloria Färber, Assad Haneya, Daniel Zimpfer, Gaik Nersesian, Ilija Djordjevic, Diyar Saeed, Finn Gustafsson, Anna M. Scandroglio, Bart Meyns, Steffen Hofmann, Jan Belohlavek, Jan Gummert, Pia Lanmueller, Evgenij V. Potapov, Mehmet Oezkur

**Affiliations:** ^1^ Department of Cardiac and Vascular Surgery University Medical Center of the Johannes Gutenberg University Mainz Mainz Germany; ^2^ Department of Cardiothoracic and Vascular Surgery Deutsches Herzzentrum der Charité (DHZC) Berlin Germany; ^3^ Department of Cardiovascular Surgery Charité – Universitätsmedizin Berlin Berlin Germany; ^4^ Heart and Diabetes Center, North Rhine‐Westphalia Bad Oeynhausen Germany; ^5^ Department of Cardiovascular Surgery University Heart Center Hamburg Hamburg Germany; ^6^ Department of Cardiac Surgery Schüchtermann Clinic Bad Rothenfelde Germany; ^7^ Department of Cardiac Surgery Heidelberg University Hospital Heidelberg Germany; ^8^ Institute of Clinical and Experimental Medicine Prague Czech Republic; ^9^ Second Department of Internal Medicine, Cardiovascular Medicine, General Teaching Hospital and 1^st^ Faculty of Medicine Charles University Prague Czech Republic; ^10^ Department of Cardiac Surgery University Hospitals Leuven Leuven Belgium; ^11^ Department of Anesthesia and Intensive Care IRCCS San Raffaele Scientific Institute Milan Italy; ^12^ Deparment of Cardiac Surgery, IRCCS Bologna St. Orsola University Hospital Bologna Italy; ^13^ Department of Surgical Sciences University of Turin Turin Italy; ^14^ Department of Cardiology University Medical Center Utrecht Utrecht Netherlands; ^15^ Department of Cardiothoracic Surgery Rigshospitalet Copenhagen Denmark; ^16^ Department of Cardiovascular Surgery University Hospital Duesseldorf Duesseldorf Germany; ^17^ Department of Cardiothoracic Surgery University Hospital RTWH Aachen Aachen Germany; ^18^ Department of Cardiac Surgery Leipzig Heart Center Leipzig Germany; ^19^ Department of Cardiac Surgery University of Essen Essen Germany; ^20^ Department of Cardiothoracic Surgery Jena University Hospital Jena Germany; ^21^ Department of Cardiovascular Surgery University Hospital Schleswig‐Holstein Kiel Germany; ^22^ Department of Surgery, Division of Cardiac Surgery Medical University of Vienna Vienna Austria; ^23^ Department of Cardiothoracic Surgery University Hospital Cologne Cologne Germany; ^24^ DZHK (German Centre for Cardiovascular Research), partner site Berlin Berlin Germany

**Keywords:** Microaxial flow pump, Durable mechanical circulatory support, Cardiogenic shock, Impella, Left heart failure

## Abstract

**Aims:**

Patients with acute decompensated advanced heart failure requiring left ventricular assist device (LVAD) implantation often experience progressive cardiac function deterioration, negatively impacting surgical outcomes. This study aimed to assess the efficacy of different microaxial flow pump (mAFP) support devices (Impella®) in achieving optimal left ventricular unloading for preconditioning and facilitating definitive treatment in this high‐risk patient cohort.

**Methods and results:**

A retrospective analysis was conducted across 19 high‐volume European centres. The study population included patients transitioning from temporary to durable circulatory support over a 7.5‐year period, with a median follow‐up of 1 year. Patients were categorized based on mAFP support capacity: those receiving high‐flow support (>5 L/min, ‘5+’) and those with lower‐flow support (3.5 L/min, ‘CP’). Patients who were initially treated with CP but subsequently upgraded to 5+ support were classified in the 5+ group. Demographic and clinical characteristics, mobilization, right heart function, and organ dysfunction outcomes were analysed. A total of 339 patients received preoperative mAFP support prior to LVAD implantation. The 5+ group comprised 247 patients (73%), including 38 patients who were upgraded from CP, while the CP group included 92 patients (27%). Baseline demographic and clinical characteristics were comparable between groups, except for mobilization status, which showed significant differences (*P* < 0.001). Patients in the 5+ group achieved higher rates of full and partial mobilization compared to the CP group. Extracorporeal life support (ECLS) was more frequently required in the CP group than in the 5+ group (40.5% vs. 33.8%; *P* < 0.001). Additionally, right ventricular assist device (RVAD) implantation was significantly more common in the CP group (29.2% vs. 18.2%; *P* = 0.026). Patients in the 5+ group demonstrated greater reductions in both vasoactive inotropic scores (*P* = 0.006) and inotropic scores (*P* = 0.008). Furthermore, liver dysfunction (*P* = 0.016), renal failure (*P* = 0.041), and the need for dialysis (*P* = 0.013) were significantly more prevalent in the CP group. There were no significant differences between the two groups in terms of LVAD operative duration (*P* = 0.637) or cardiopulmonary bypass time (*P* = 0.408).

**Conclusions:**

High‐flow mAFP devices (+5) provided superior haemodynamic support, enhanced left ventricular unloading, and reduced dependence on catecholamines compared to lower‐flow CP devices. These improvements were associated with lower rates of right ventricular failure, renal dysfunction, and liver injury. However, no statistically significant difference was observed between mAFP groups regarding 30‐day mortality rates.

## Graphical abstract

In the context of patients with severe heart failure, it is crucial to ensure adequate preconditioning before considering the implantation of dLVAD. Although several types of mechanical circulatory assist devices are available and all offer a significant advantage, the present study suggests that the higher the flow rate provided by the Impella®, the better the postoperative outcomes with reduced complications. This study highlights the benefits of mAFP devices delivering more than 5 L flow per minute, not only in terms of haemodynamic considerations but also in relation to postoperative outcomes with concomitant LVAD implantation. This underscores the importance of considering mAFP upgrades for patients who were initially treated with Impella CP.

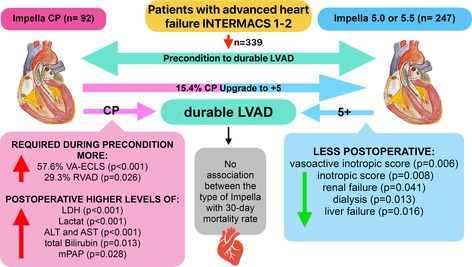

## Introduction

Cardiogenic shock (CS) remains a challenge in cardiovascular medicine with 30‐day mortality rates of 40 to 50%^1^ in patients requiring mechanical circulatory support.^2^ One therapeutic option for these patients are durable left ventricular assist devices (dLVADs). Due to the shortage of available heart donors, there has been a significant rise in the utilization of dLVADs, not only as a bridge to transplantation but also as a long‐term solution.^3^

Elective dLVAD implantation substantially lowers the risk of acute adverse events such as right ventricular failure and mortality when compared to acute heart failure and shock patients.^4^ In consequence, achieving adequate compensation with the support device prior to surgery is crucial for optimizing outcomes in these patients.^5^

Patients with severe CS may have higher support requirements and intrinsically higher mortality rates due to their clinical presentation.^6^ The increasing use of microaxial flow pumps (mAFP) (Impella® Abiomed, Danvers, Massachusetts, USA) for the support of patients with CS indicates their success in clinical practice.^2^, ^7^ The ability to rapidly reduce ventricular wall stress and myocardial oxygen consumption, increase antegrade flow, and consequently reduce ventricular pressure and volume facilitates the reversal of organ damage.^8^ In addition, the extended durability of mAFP devices, along with their potential to serve as a weaning tool for patients on extracorporeal life support (ECLS), contributes to the reduction of ECLS associated complications. This, in turn, has a positive impact on outcomes following dLVAD implantation.^9^

While it has been demonstrated that the outcomes and complications observed in patients requiring stabilization with mechanical circulatory support are comparable across different types of support,^10^ addressing the need to minimize complications such as bleeding and improve both short‐ and long‐term survival rates remains essential.^11^

Different versions of mAFP vary in implantation site and the blood flow level provided. For patients who have severely compromised left ventricular (LV) and end‐organ functionality, high pump flows of up to 5.5 L/min can effectively relieve congestion in the lungs, right ventricle, and other vital organs such as the liver and kidneys. Ideally, this leads to improved outcomes with the subsequent implantation of an LVAD in patients with severe heart failure with reduced ejection fraction and impaired end‐organ function during the pre‐shock phase.^12^

This study systematically reviewed and compared the clinical outcomes, haemodynamic stabilization, and device‐related complications associated with various mAFP used as bridges to dLVADs.

## Patients and methods

### Patient population and data collection

The present study is a subanalysis of retrospective international multicentre registry data from 19 European high‐volume centres.^5^ The study examined patients transitioning from different mAFP to dLVADs over a 7.5‐year period from January 2015 to July 2022. Patients were predominantly male (*n* = 285; 83.5%) and supported with a mAFP for an average of 9.00 [5.00, 14.00] days. Patients supported with the mAFP 2.5 were excluded from the evaluation because of its reduced utilization following the introduction of the mAFP CP.^13^ Additionally, patients who received a total artificial heart or biventricular assist device implantation (such as percutaneous biventricular support with two mAFP or RVAD with mAFP) were also excluded from the analysis. All patients were INTERMACS Level 1–2 prior to mAFP implantation.

Preceding the implantation of the dLVAD, all patients underwent haemodynamic stabilization utilizing mAFP CP/5.0/5.5 devices. The selection of mAFP support in our study cohorts varied and was primarily determined by availability at the clinical centre and the urgency of haemodynamic stabilization for the patient. Among the available options, mAFP CP was the most accessible choice.^14^ In certain instances, these support systems were combined with venoarterial extracorporeal life support (va‐ECLS), known as the ECMELLA approach,^15^, ^16^, ^17^ to ensure effective unloading of the LV and facilitate pulmonary decongestion or gradual weaning of patients from ECLS.^18^ mAFP CP devices were implanted percutaneously via puncture of the femoral artery, while 5.0 and 5.5 devices were inserted either through the axillary artery or from the ascending aorta using a vascular prosthesis.

No standardized protocol has been established for the process of weaning from mAFP or for the implantation of LVAD. The selection of patients who qualified for LVAD implantation and the determination of optimal timing was therefore at the discretion of the providing medical centre. Haemodynamic stabilization emerged as a key consideration in the decision‐making process.

The study also observed the functional status of patients while receiving mAFP support, their requirement for additional mechanical cardiac support during mAFP support, and their status after discharge. Catecholamine requirements were quantified using the vasoactive‐inotropic score and inotropic score and haemolysis indicators (a biochemical marker) were collected. Haemodynamic parameters included mean pulmonary artery pressure (mPAP), pulmonary artery pulsatility index (PAPi), and central venous pressure. Covariates missing more than 50% of values were excluded from the analysis.

### Study outcome

The primary endpoint for the study was the haemodynamic effect provided by each device and assessment of their impact on catecholamine requirement before the dLVAD implantation. Secondary endpoints include short‐term mortality (30 days) and complications after LVAD implantation (surgery time, bleeding disorders, thrombosis, infection, organ failure).^19^

### Statistical analysis

Statistical analysis was performed using the Statistical Package for the Social Sciences (SPSS) for macOs® version 29.0 (SPSS Inc., Chicago, Illinois, USA). All data were anonymized and treated according to data protection regulations: EU Data Protection Directive (95/46/EC) I Data Protection 2020.

Continuous variables were presented as mean ± standard deviation, or median (interquartile range) in the case of non‐normal data. Due to similarities in implantation technique and flow, the mAFP with 5.0 and 5.5 L/min flow were merged into one group especially since the 5.5 L/min mAFP is the updated version of the 5.0 L/min. All statistical analyses were performed comparing the mAFP CP (3.5 L/min) and mAFP versions, which provide more than 5.0 L/min (5+). Categorical variables were shown as absolute (*n*) and relative (%) frequencies. Continuous variables with a normal distribution were tested using Student's *t*‐test, while those that were not normal were tested using Mann–Whitney test. The non‐parametric or categorical data were tested with Pearson's chi‐square test and Fisher's exact test. The haemodynamic differences between each mAFP device were assessed using the Kruskal–Wallis H test, which was also performed to evaluate the difference between the values of liver function and metabolic parameters between each mAFP. Retrospective examination of patient data was performed using the Model of End‐Stage Liver Disease (MELD) and MELD excluding international normalized ratio (MELD‐XI) score as a predictor of mortality.

To mitigate the potential impact of selection bias and reduce variability within both groups, a series of analyses were performed on selected patients who received mAFP support with 3.5 L/min (CP) and those who received support with more than 5.0 L/min (5+). Adjustment for differences between patients with support with mAFP CP or 5+ was made using propensity scores, and corresponding stabilized inverse probability of treatment weights (IPTW) were calculated using a binary logistic regression model. The logistic regression for mAFP utilization was calculated considering covariates such as age, sex, body mass index (BMI), body surface area, history of prior cardiac surgery, history of previous stroke, aetiology of CS, and concomitant diseases (atrial fibrillation, diabetes, peripheral arterial disease, presentation after cardiopulmonary resuscitation) (*Table* *1*).

**Table 1:** Demographics and clinical characteristics for each mAFP group

Clinical characteristics	Total (*n* = 339)	CP (*n* = 92)	5+ (*n* = 247)	*P* value
		27%	73%	
Age (years)	55.13 ± 12.57	55.01 ± 11.62	55.18 ± 12.93	0.906
Sex				0.357
Male	283 (83.5)	74 (80.4)	209 (84.6)	
Female	56 (16.5)	18 (19.6)	38 (15.4)	
Mean BMI (kg/m^2^)	26.99 ± 4.86	26.31 ± 4.15	27.24 ± 5.08	0.090
Mean BSA (kg/m^2^)	2.35 ± 2.52	2.66 ± 3.61	2.23 ± 1.94	0.293
AMI	107 (31.6)	33 (35.9)	74 (30.0)	0.807
ICM decompensated	96 (28.3)	21 (22.8)	75 (30.4)	
DCM decompensated	92 (27.1)	26 (28.3)	66 (26.7)	
Fulminant myocarditis	26 (7.7)	7 (7.6)	19 (7.7)	
Another cardiomyopathy	11 (3.2)	3 (3.3)	8 (3.2)	
other aetiology	7 (2.1)	2 (2.2)	5 (2.0)	
CPR before Impella support	102 (30.1)	38 (41.3)	64 (26.3)	*0.008*
Previous cardiac surgery	56 (16.5)	11 (12)	56 (17.7)	0.088
Previous stroke	36 (11.4)	11 (12)	25 (11.2)	0.850
Atrial fibrillation	127 (37.5)	29 (31.5)	127 (37.5)	0.168
Diabetes mellitus	100 (29.5)	24 (26.1)	76 (30.8)	0.401
Peripheral arterial disease	30 (8.8)	12 (13)	18 (7.3)	0.097
No mobilization	111 (32.7)	57 (62)	54 (25.1)	*<0.001*
Mobilization in bed	96 (28.3)	29 (31.5)	67 (31.2)	
Mobilization to the bedside	41 (12.1)	0	41 (19.1)	
Mobilization out of bed	43 (12.7)	6 (6.5)	37 (17.2)	
Mobilization out of room	16 (4.7)	0	16 (7.4)	

AM, acute myocardial infarction; BMI, body max index; BSA, body surface area; CPR, cardiopulmonary resuscitation; DCM, dilated cardiomyopathy; ICM, ischaemic cardiomyopathy.

Categorical variables are expressed as *n*/total (%), and continuous variables are expressed as mean ± standard deviation.

Values were considered statistically significant at a probability factor *P* ≤ 0.05.

To evaluate the secondary endpoints, analysis of the cumulative mortality for the first 30 days was performed using the Kaplan–Meier method, with the log‐rank test used for the comparison between the two groups. In addition to the test results, effect estimates with 95% confidence intervals are given. Median and the 25–75 percentile were calculated as a reflection of the effect of each mAFP treatment on laboratory parameters. Values were considered statistically significant at a probability factor *P* ≤ 0.05.

### Ethics

The study protocol was approved by the individual Health Research Ethics Boards (EA2/196/21) on 16 July 2023. Patients provided informed written consent for the use of their pseudonymized data for research purposes. The need for informed written consent for the publication of their study data in this specific publication was waived by the Health Research Ethics Boards and conform with the Helsinki Declaration.

## Results

From January 2015 to July 2022, a total of 339 patients underwent surgical implantation of different mAFP as transition to dLVADs. The median age was 55.13 ± 12.57 years, with 283 (83.5%) of patients being male. Of the 339 patients who underwent implantation with CP, 5.0, or 5.5 prior to dLVAD, a total of 92 patients (27%) were initially implanted with 5.5 (27%), 155 patients with 5.0 (45.5%) and 92 patients with CP (27%). Most patients presented with severe CS caused by acute myocardial infarction (31.6%), followed by acute decompensation of ischaemic cardiomyopathy (28.3%) and dilated cardiomyopathy (27.1%) (*Table* *1*).

After correction with logistic regression, there were no relevant differences in pre‐existing diseases between patients receiving mAFP CP and mAFP 5+, except for fewer patients with history of peripheral vascular disease in the mAFP CP group (*Table* *2*). After IPTW adjustment, the survival was similar between the groups (OR: 1.34, 95% CI: [0.58–3.07], *P* = 0.48).

**Table 2:** Preoperative patient characteristics according to logistic regression

Patient's characteristics	Odds ratio with (95% CI)	Lower	Upper	*P* value
Age (years)	0.985	0.953	1.010	0.243
Sex male	1.318	0.517	2.618	0.430
Mean BMI (kg/m^2^)	1.041	0.971	1.103	0.167
Mean BSA (kg/m^2^)	0.954	0.825	1.046	0.318
Previous cardiac surgery	1.479	1.019	3.259	0.331
Previous stroke	0.759	0.258	1.739	0.514
AMI				0.971
ICM decompensated	1.188	0.582	2.425	0.635
DCM decompensated	0.878	0.409	1.885	0.739
Fulminant myocarditis	0.875	0.294	2.601	0.810
Another cardiomyopathy	1.034	0.229	4.664	0.965
Other aetiology	0.631	0.100	3.973	0.624
Atrial fibrillation	1.446	0.776	2.615	0.254
Diabetes mellitus	1.097	0.743	2.708	0.289
Peripheral arterial disease	0.268	0.140	0.891	***0.027***
CPR before Impella support	0.873	0.329	1.028	0.062

AMI, acute myocardial infarction; BMI, body max index; BSA, body surface area; CPR, cardiopulmonary resuscitation; DCM, dilated cardiomyopathy; ICM, ischaemic cardiomyopathy.

Values were considered statistically significant at a probability factor *P* ≤ 0.05.

Baseline and clinical characteristics were compared between patients who received support with mAFP CP and 5+ (*Table* *1*). No statistically significant difference was observed between the utilization of different types of mAFP support and the aetiology of cardiogenic shock. It was observed that a majority of patients who experienced cardiopulmonary resuscitation before the initiation of mAFP support exhibited a higher frequency of stabilization with mAFP CP, reaching 41.3%. In contrast, patients stabilized with a mAFP 5+ accounted for 26.3%, indicating a statistically significant difference (*P* = 0.008). In relation to the site of mAFP implantation, a statistically significant increase in mobility was observed in patients who were administered mAFP 5+ support (*P* < 0.001).

**Figure 1:**
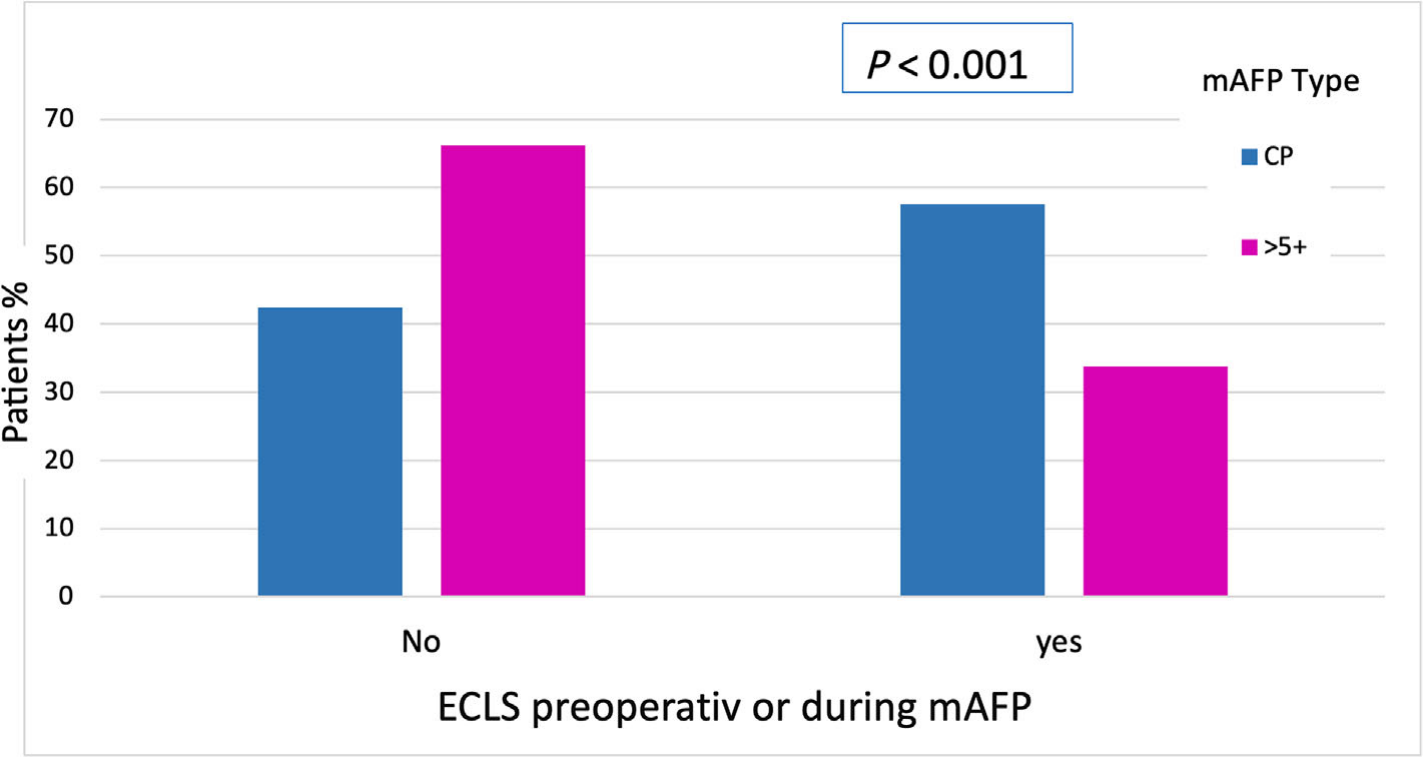
Use of extracorporeal life support pre‐ or post‐implantation mAFP. ECLSpre/durImp, extracorporeal life support preoperative/during Impella support; mAFP, microaxial flow pump.

Additionally, 132 (40.5%) patients received ECLS before or in addition to mAFP, with a median time on ECLS of 7.00 (4.00, 11.00) days (*Figure* *1* and *Table* *3*). Patients who underwent preconditioning with mAFP CP received prior or combined ECLS in a significantly higher proportion with 57.6% compared to those who received mAFP 5+ support 33.8% (*P* < 0.001). Intra‐aortic balloon pumps were inserted in 31 (9.1%) patients before implantation of a mAFP device. Thirty‐eight (15.4%) patients with mAFP 5+ support was previously treated with mAFP CP and then switched to 5+ due to insufficient circulatory support (*Table* *3*).

**Table 3:** Mechanical circulatory support for each mAFP group

mAFP	Total (*n* = 339)	CP (*n* = 92)	>5+ (*n* = 247)	*P* value
mAFP duration (days)	9.00 [5.00, 14.00]	8.00 [5.00, 10.75]	7.50 [4.00, 18.25]	*<0.001*
mAFP upgrade	38 (14.7)	0	38 (15.4)	*<0.001*
IABP pre mAFP	31 (9.1)	8 (8.7)	23 (9.3)	0.861
RVAD during mAFP	72 (21.2)	27 (29.3)	45 (18.2)	*0.026*
RVAD support duration (days)	17,50 [10.00, 23.75]	15,00 [10.75, 22.25]	11.50 [2.75, 21.25]	0.877
ECLS pre or during mAFP	132 (40.5)	53 (57.6)	79 (33.8)	*<0.001*
ECLS duration (days)	7.00 [4.00, 11.00]	7.00 [3.75, 8.50]	7.00 [2.75, 11.25]	0.502
IS	3.60 [0.00, 7.73]	7.29 [1.15, 9.23]	2.65 [0.00, 8.02]	*0.008*
VIS	3.65 [0.00, 7.79]	7.62 [1.15, 9.84]	3.77 [0.00, 8.02]	*0.006*
MELD	14.00 [10.00, 22.75]	17.00 [11.00, 24.50]	14.00 [9.00, 22.00]	0.088
MELDXI	14.00 [10.25, 21.00]	17.50 [11.00, 23.00]	13.00 [10.00, 20.00]	*0.014*

ECLS, extracorporeal life support; IABP, intra‐aortic balloon pump; IS, inotropic score; LVAD, left ventricular assist device; mAFP, microaxial flow pump; MELD, Model of End‐Stage Liver Disease; MELD‐XI, MELD excluding international normalized ratio; RVAD, right ventricular assist device; VIS, vasoactive inotropic score.

Categorical variables are expressed as *n*/total (%), and continuous variables are expressed as median (interquartile range).

Values were considered statistically significant at a probability factor *P* ≤ 0.05.

**Figure 2:**
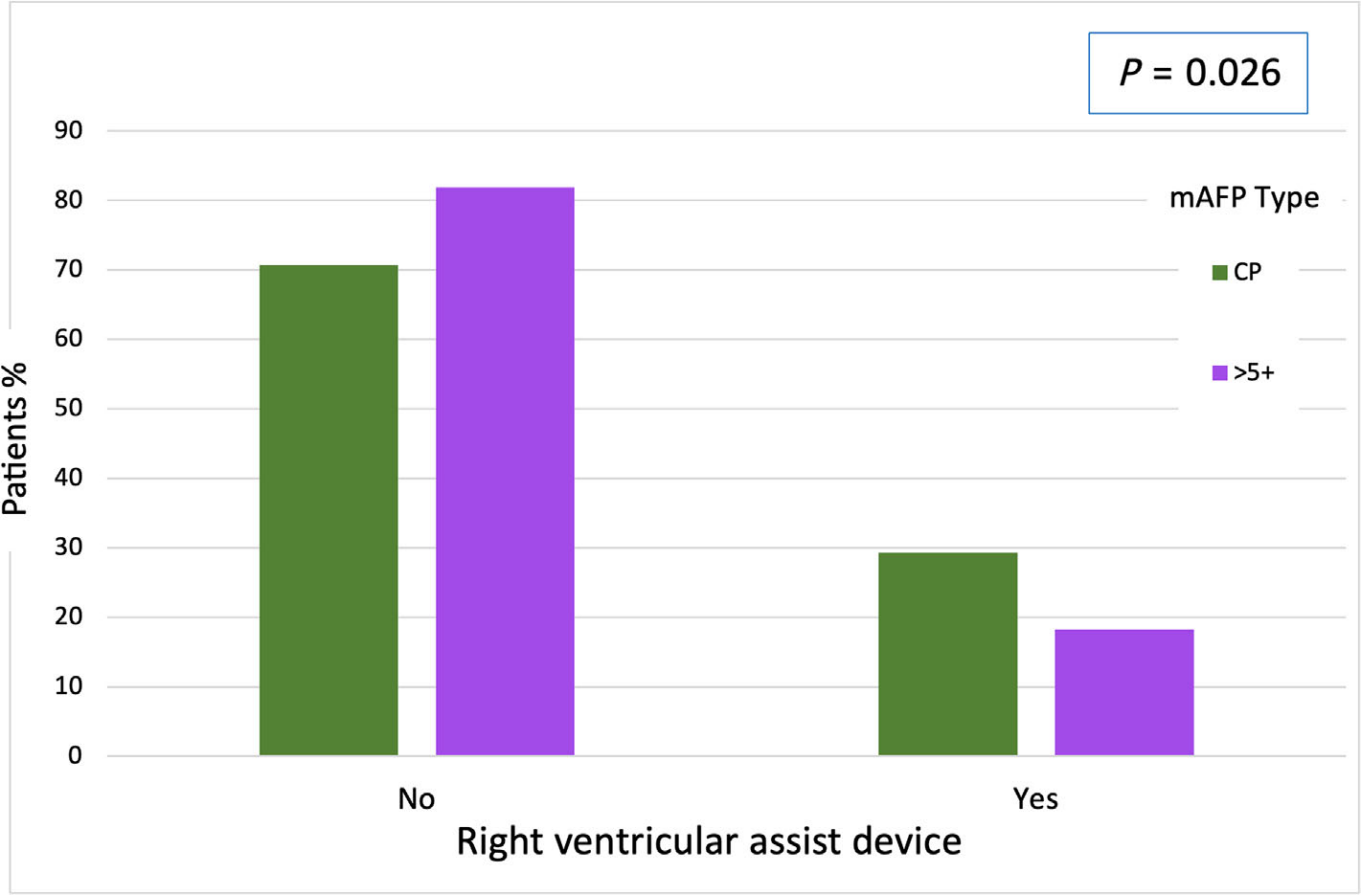
RVAD support after dLVAD implantation. dLVAD, durable left ventricular assist device; mAFP, microaxial flow pump; RVAD, right ventricular assist device.

Following a median duration of mAFP support of 9.00 (5.00, 14.00) days, patients underwent transition to one of the dLVADs available: HeartMate 3 (*n* = 204), HeartWare (*n* = 128), or EXCOR (*n* = 7). Additionally, 72 patients (21.2%) experienced perioperative right heart failure and required temporary right ventricular assist device (RVAD) implantation. Patients who require a RVAD due to right heart failure, were significantly higher in the CP group with 29.2% then in the 5+ group with 18.2% (*P* = 0.026; *Figure* *2* and *Table* *3*). Patients who required RVAD support were assisted for a median duration of 17.50 (10.00, 23.75) days.

There was a significant association between the type of mAFP and the use of catecholamines, indicating that a higher the flow rate offered by the mAFP led to lower inotropic and vasoactive‐inotropic scores (*P* = 0.008 and *P* = 0.006, respectively) (*Table* *3*).

No statistically significant differences were observed between the mAFP groups regarding the duration of LVAD implantation or time on cardiopulmonary bypass (*Table* *4*). Likewise, the characteristics of the mAFP do not affect the incidence of stroke. However, a subanalysis for stroke outcomes from the same study showed that multiple factors were associated with haemorrhagic stroke, suggesting a proactive treatment target to minimize this complication.^20^

**Table 4:** LVAD Implantation and postoperative complications for each mAFP group

Postoperative complications	Total (*n* = 339)	CP (*n* = 92)	5+ (*n* = 247)	*P* value
		27%	73%	
CPB time LVAD implantation (min)	80.50 [0.00, 120.00]	89.00 [0.00, 121.00]	80.00 [0.00, 120.00]	0.637
Surgery time LVAD Implantation (min)	239.00 [180.00, 293.00]	241.50 [199.00, 284.00]	241.50 [170.75, 300.00]	0.408
Rethoracotomy	79 (23.3)	24 (26.1)	55 (22.3)	0.459
Stroke	62 (18.3)	20 (21.7)	42 (17)	0.316
Driveline infection	81 (24.5)	24 (26.7)	57 (23.8)	0.583
Gastrointestinal bleeding	46 (13.6)	12 (13)	34 (13.8)	0.863
Pump thrombosis	20 (6)	3 (3.3)	17 (7)	0.205
Postoperative renal failure	109 (41.3)	37 (51.4)	72 (37.5)	***0.041***
Postoperative dialysis	110 (38.7)	38 (50.7)	72 (34.4)	***0.013***
Postoperative liver failure	47 (18.3)	19 (27.9)	28 (14.8)	***0.016***
Postoperative respiratory failure	105 (38)	32 (42.7)	73 (36.3)	0.334
LVAD mortality	117 (34.5)	37 (40.2)	80 (32.4)	0.178
Mortality 30 days	43 (12.7)	14 (15.2)	29 (11.7)	0.392

CPB, cardiopulmonary bypass; LVAD, left ventricular assist device; mAFP, microaxial flow pump.

Categorical variables are expressed as *n*/total (%), and continuous variables are expressed as median (interquartile range).

Values were considered statistically significant at a probability factor *P* ≤ 0.05.

Patients who underwent stabilization with lower‐flow mAFP showed a higher incidence of postoperative liver dysfunction (CP: 27.9% vs. 5+: 14.8, *P* = 0.016) and renal failure (CP: 51.4% vs. 5+: 37.5, *P* = 0.041) assisted with dialysis post LVAD implantation (CP: 50.7% vs. 5+:34.4, *P* = 0.013). In contrast, postoperative complications such as bleeding or infection did not show a statistically significant association with the type of mAFP used as preoperative preconditioning (*Table* *4*).

**Figure 3:**
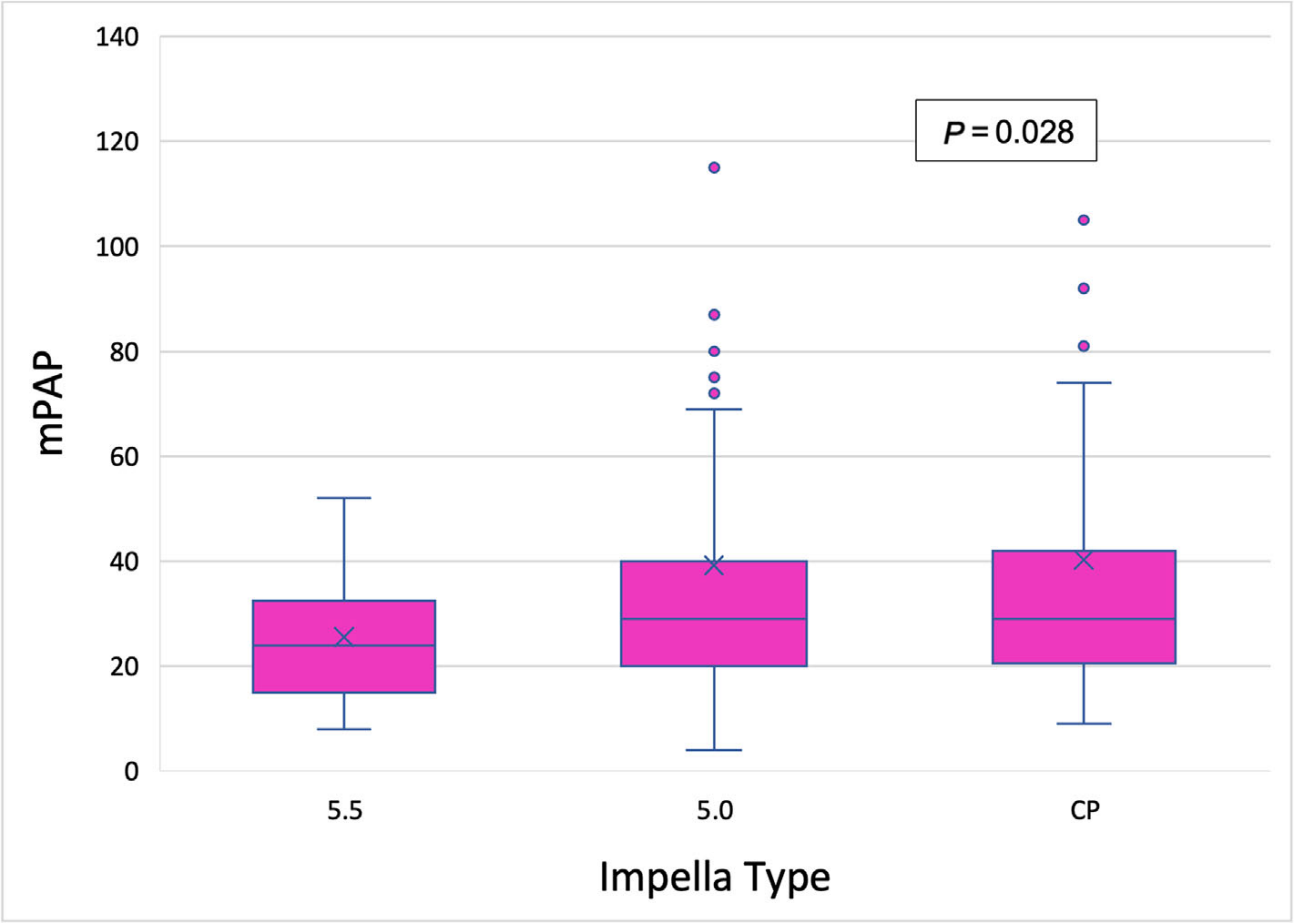
Kruskal–Wallis test for mPAP value between different microaxial pumps. mPAP, mean partial arterial pressure.

**Figure 4:**
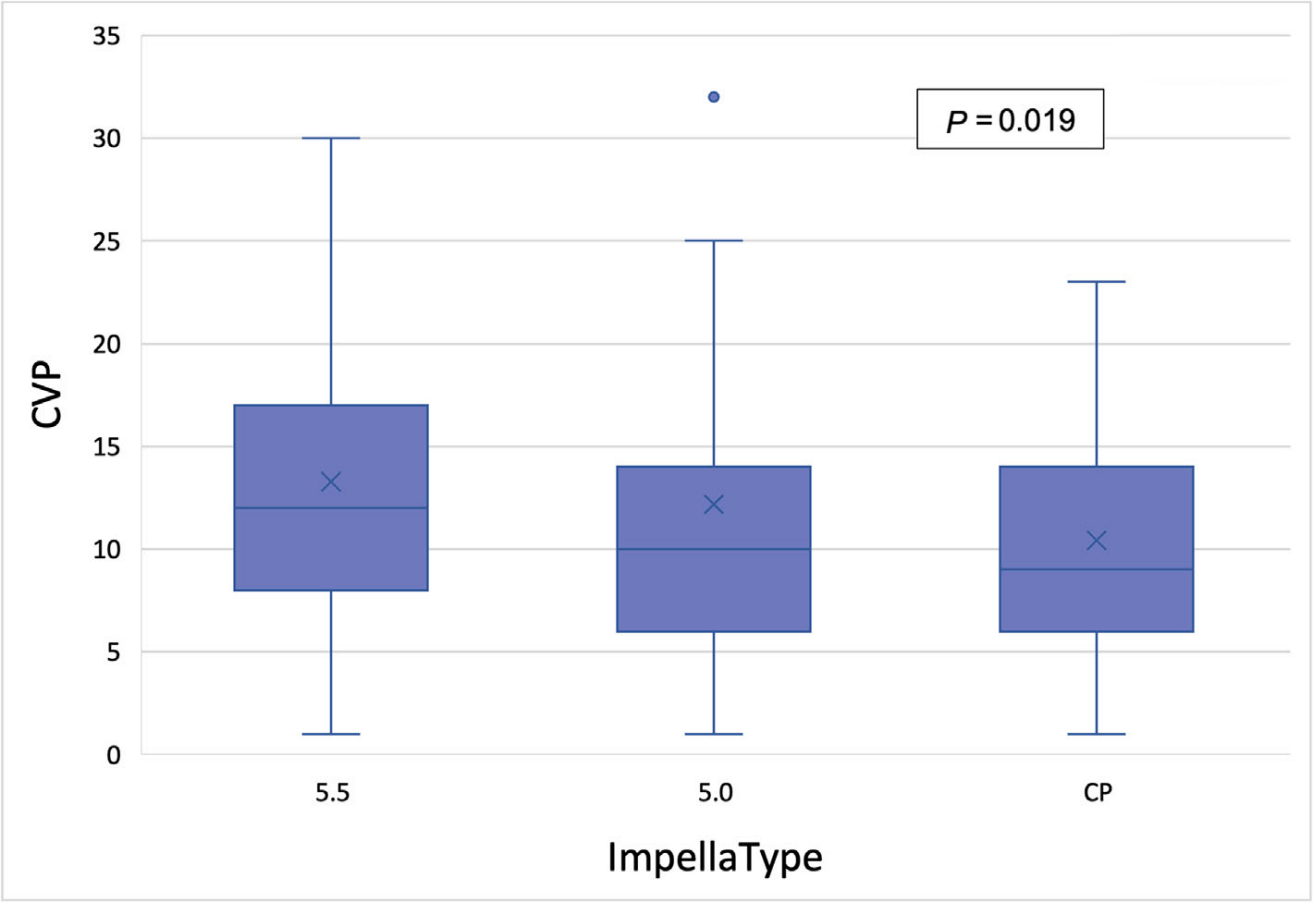
Kruskal–Wallis test for CVP value between different microaxial pumps. CVP, central venous pressure.

**Figure 5:**
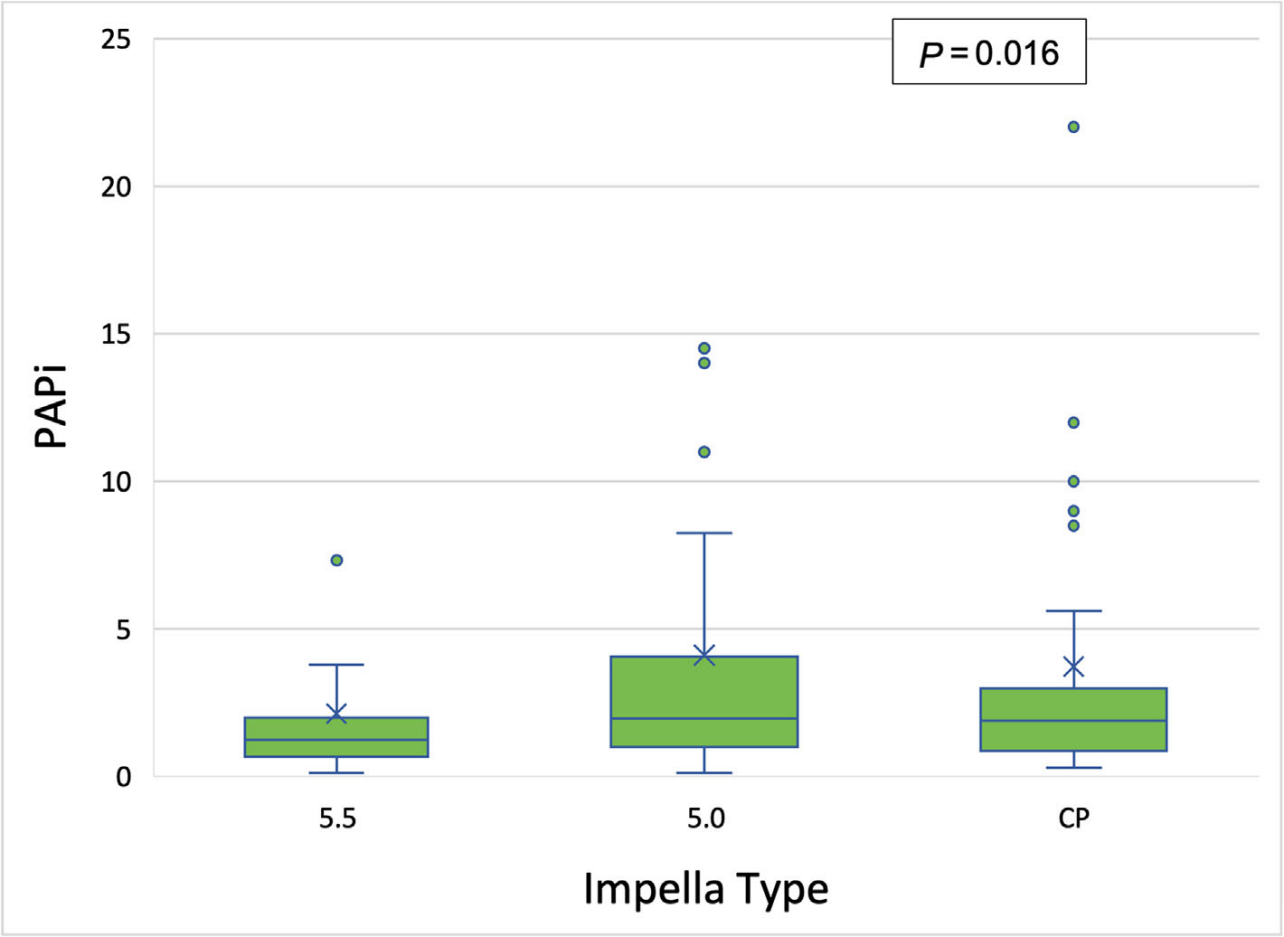
Kruskal–Wallis test for PAPi value between different microaxial pumps. PAPi, pulmonary artery pulsatility index.

Our analysis revealed a statistically significant association between haemodynamic parameters, such as mPAP (*P* = 0.028), central venous pressure (CVP) (*P* = 0.019), PAPi (*P* = 0.016), and the type of mAFP support used (*Table* *5* and *Figures* *3*, *4*, *5*). Patients with 5.5 showed a significantly reduced mPAP in comparison with the other treatment groups (*Figure* *3*). The median PAPi was 2.6 ± 3.0 and was higher among patients who received CP preconditioning (3.2 ± 4.2) compared to those who received 5.0 (2.9 ± 2.8) or 5.5 (1.6 ± 1.5) (*Figure* *5*).

**Table 5:** Hemodynamic parameters for each mAFP group

Haemodynamic parameters	Total (*n* = 339)	5.5 (*n* = 92)	5.0 (*n* = 155)	CP (*n* = 92)	*P* value
mPAP	31.36 ± 19.10	24.80 ± 11.14	33.37 ± 20.02	35.74 ± 23.25	***0.028***
CVP	11.13 ± 5.96	12.88 ± 6.33	10.65 ± 5.83	9.76 ± 5.23	***0.019***
PAPi	2.62 ± 2.98	1.64 ± 1.48	2.96 ± 2.87	3.23 ± 4.23	***0.016***

Abbreviations: CVP, central venous pressure; mAFP, microaxial flow pump; mPAP; mean pulmonary artery pressure; PAPi, pulmonary artery pulsatility index.

Categorical variables are expressed as *n*/total (%), and continuous variables are expressed as mean ± standard deviation.

Values were considered statistically significant at a probability factor *P* ≤ 0.05.

Liver enzymes such as aspartate transferase, alanine transaminase, and total bilirubin showed a statistically significant difference according to the type of mAFP used. Notably, patients who received mAFP 5+ support exhibited the lowest levels of aspartate transferase, alanine transaminase, and total bilirubin among the groups studied (*Table* *6*).

**Table 6:** Postoperative laboratory parameters (median, 25th and 75th percentiles)

Laboratory parameters	Total (*n* = 339)	CP (*n* = 92)	5+ (*n* = 247)	*P* value
		27%	73%	
Hb	9.5 (8.5, 10.5)	9.7 (9.0, 10.8)	9.7 (8.7, 10.6)	***0.045***
BE	0.2 (−1.9, 2.3)	−0.1 (−2.1, 1.4)	0.5 (−1.5, 3.0)	0.290
pH	7.43 (7.38, 7.47)	7.43 (7.38, 7.48)	7.43 (7.39, 7.47)	0.966
LDH	607 (443.5, 992.5)	707.5 (558.2, 1400.3)	510.5 (375.0, 769.0)	***<0.001***
Lactate	1.1 (0.8, 1.5)	1.3 (0.9, 1.72)	1.1 (0.8, 1.4)	***<0.001***
AST	63.0 (38.0, 133.0)	102.5 (51.0, 191.7)	48.0 (31.0, 89.8)	***<0.001***
ALT	53.0 (30.0,115.0)	99.0 (47.0,183.5)	46.4 (27.7, 83.0)	***<0.001***
GGT	101.0 (54.0–206.5)	100.8 (49.7, 214.4)	105.5 (59.0, 197.2)	0.458
Total bilirubin	1.3 (0.8–2.6)	1.8 (0.9, 3.8)	1.3 (0.7, 2.6)	***0.013***
Urea	48.2 (32.0–76.9)	48.8 (33.0, 73.2)	47.5 (31.2,72.2)	0.602
Creatinine	1.1 (0.81–1.)	1.1 (1.8, 1.9)	1.0 (0.8, 1.6)	0.356

ALT, alanine transaminase; AST, aspartate transferase; BE, base excess; GGT, gamma‐glutamyl transferase; Hb, haemoglobin; LDH, lactate dehydrogenase; mAFP, microaxial flow pump.

Categorical variables are expressed as *n*/total (%), and continuous variables are expressed as mean ± standard deviation.

Values were considered statistically significant at a probability factor p ≤ 0.05.

**Figure 6:**
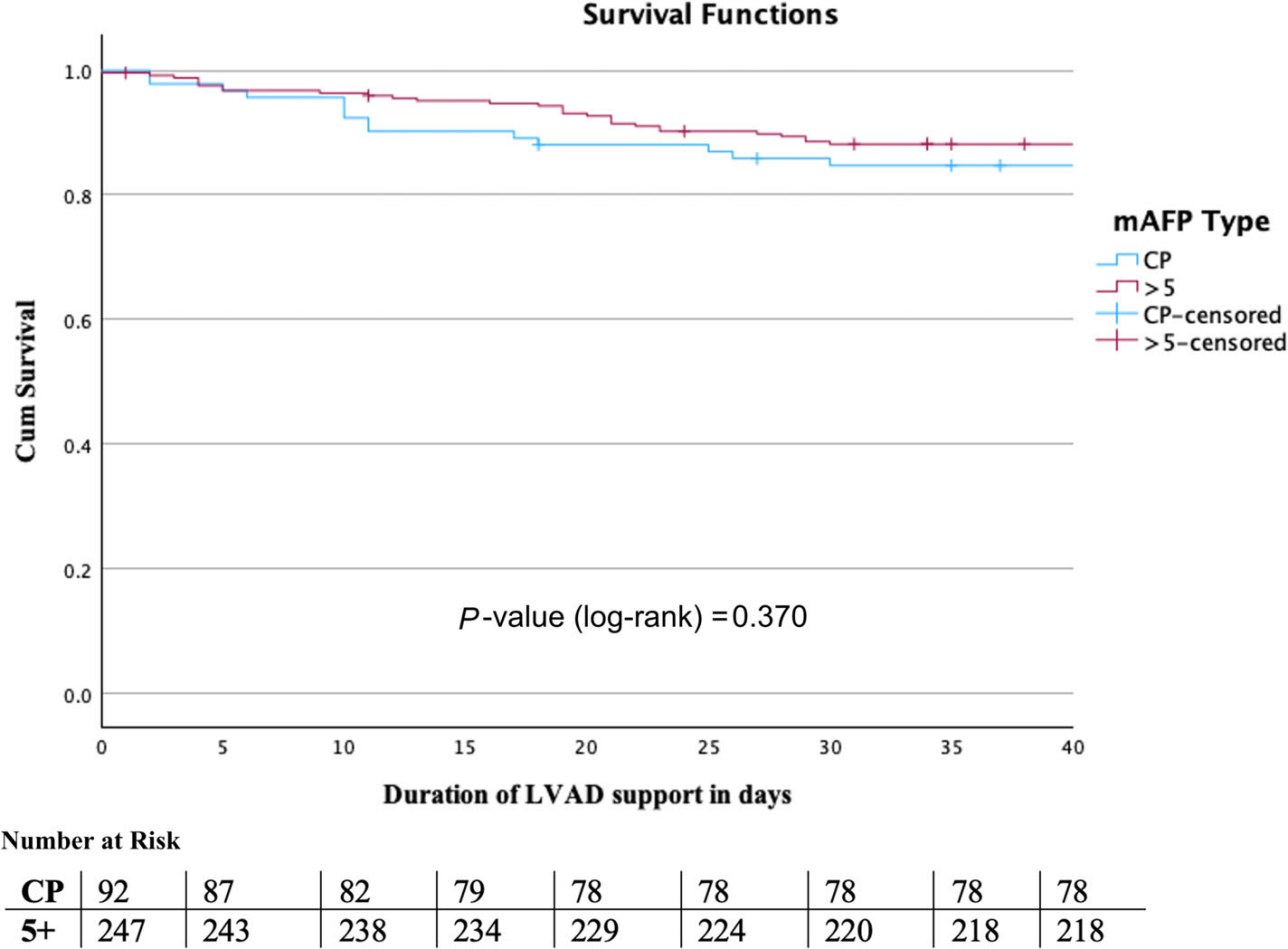
Kaplan–Meier estimates for 30‐day survival for different microaxial flow pumps. LVAD, left ventricular assist device, mAFP, microaxial flow pump.

There was no significant difference in 30‐day mortality between the two mAFP groups (*P* = 0.392; *Table* *4*). The 30‐day mortality was 15.2% in the mAFP CP group and 11.7% in the mAFP 5+ group. Kaplan–Meier survival curves showed a slight trend towards higher survival in the mAFP 5+ group compared to the mAFP CP group; however, no statistically significant difference in survival between both groups was observed at day 30 (*P* = 0.370) (*Figure* *6*). Furthermore, it was observed that despite concomitant support with ECLS or RVAD, no significant difference in 30‐day mortality was evident (with ECLS *P* = 0.325, with RVAD *P* = 0.793). While no significant differences were found in the three analyses, there was a trend towards better survival in those patients who had received support with mAFP providing a flow greater than 5 L/min (*Figure* *6*).

## Discussion

This retrospective study showed clear clinical advantages of complete LV unloading by high‐flow Impella 5+ over Impella CP during optimization of patients for bridging to dLVAD. CS remains a primary cardiovascular disorder with extremely high in‐hospital mortality rates.^14^ Given the necessity of definitive treatment such as dLVAD implantation or transplantation in these patients, the concept of preconditioning before these procedures has become essential.^8^ Consequently, there has been increased adoption of mAFP as a bridge to final decision on the dLVAD to implant.^18^ The choice of an appropriate mAFP is typically influenced by patient‐specific factors and conditions, as well as the availability of different devices. The mAFP has gained popularity due to its ease of access, allowing patient mobilization, optimization of right ventricular function, and offering various advantages such as promoting LV unloading and reducing wall stress associated with its distension, which are major criteria of myocardial recovery. All of these factors have demonstrated marked improvements in patient outcomes.^21^, ^22^

The study assessed which patients showed a better preconditioning status prior to dLVAD implantation. A preference for a specific type of mAFP implant emerged depending on the patient pathology. The CP device was most frequently implanted in patients with *acute myocardial infarction* (35.9%), probably due to its prompt percutaneous placement in acute emergency situations and in haemodynamically unstable patients.^13^

In contrast, for those patients with decompensated ischaemic cardiomyopathy, there was a greater preference for mAFP devices that provide higher‐flow support (30.4%), but there was no significant statistical difference in terms of the type of mAFP used and the aetiology of cardiogenic shock in our cohort. Moreover, 5.5. has been designed to provide support for an extended duration, effectively stabilizing patients experiencing LV‐dominant refractory CS.

This extended support allows enough time to perform targeted dLVAD and transplantation assessment and enables a smooth transition process.^23^

In matched patients, baseline characteristics pertinent to propensity score calculation exhibited a satisfactory equilibrium between the two patient groups. It is noteworthy, however, that individuals supported with mAFPs providing higher flow were inclined towards a heightened prevalence of comorbidities. This tendency was largely attributable to the inclusion of a majority of patients with peripheral arterial disease and those who had undergone prior cardiac surgery in this particular group.

The ECMELLA concept, which combines mAFP with va‐ECLS, was also used in multiple patients in this study. According to the advanced stage of heart failure observed in the cohort, 132 patients (40.5%) required the use of va‐ECLS before or after the implantation of a mAFP. This pathophysiological approach aims to mitigate the adverse effects associated with va‐ECLS, such as increased ventricular wall stress, and elevated myocardial oxygen consumption, which are observed when VA‐ECLS is used alone. The addition of Impella support provides the benefit of reducing left ventricular afterload and enhancing myocardial unloading.^17^, ^24^ In our study, except for one patient, all individuals had arterial lactate levels below 7.9 mmol/L. As reported by Aludaat *et al*.,^25^ patients with arterial lactate levels exceeding 7.9 mmol/L during ongoing VA‐ECLS support did not benefit from an upgrade to ECMELLA. This underscores the critical importance of arterial lactate as a marker of ischaemia and its prognostic relevance in this context.^26^

As shown in *Figure* *1*, those patients who received support with a mAFP device offering higher flow were the patients who had a lower risk for additional va‐ECLS support.^27^ Although, due to the nature of the study, the duration of ECMELLA for each patient could not be specified, it was observed that patients requiring additional support with va‐ECLS and supported with Impella CP exhibited less haemodynamic stability in the postoperative phase compared to those receiving support with Impella 5+.

Here, it is important to emphasize that the mAFP 5.5 offers the highest blood flow of all currently available pumps,^4^ and a significant decrease in inotropic and vasoactive‐inotropic scores was observed in those patients who received support with higher flow rate mAFP, allowing haemodynamic stabilization during mAFP.^12^

Patients who underwent the transition to dLVAD with CP were found to have the highest incidence of postoperative RVAD requirement. Based on these findings, it was hypothesized that patients undergoing preconditioning with mAFP with peak flow rates up to 5.0 L/min experienced greater stabilization of the right ventricle, leading to improved postoperative outcomes during the targeted dLVAD procedure. Higher‐flow mAFP 5+ has been reported in the literature to improve right ventricular and renal function by unloading the left ventricle, thereby improving forward flow and end‐organ perfusion.^28^, ^29^

These observations were corroborated by the fact that patients receiving mAFP CP preconditioning showed a higher incidence of liver failure, resulting in elevated liver function parameters such as bilirubin, AST, and AGT, compared to those not supported with mAFP up to 5.0 L/min.

The advantage of preconditioning with a mAFP for dLVAD implantation goes beyond improving the patient's haemodynamic stability and preoperative status. It also contributes to better therapeutic decision making, leading to better surgical outcomes, although these outcomes may vary depending on a diversity of other factors. Despite the multifactorial nature of postoperative complications associated with LVADs, effective haemodynamic preconditioning can enhance organ perfusion and reduce the occurrence of renal and hepatic failure, particularly in patients experiencing biventricular heart failure onset.^21^

Patients supported with the Impella 5.5 demonstrated a significant reduction in mPAP, which is an independent prognostic marker and an indicator of decreased LV filling pressure. In contrast, patients assisted with the Impella CP and 5.0 devices exhibited higher PAPi values. This may be attributed to the substantial proportion of patients in these groups who required adjunctive RV support, such as ECLS or RVAD, which likely enhanced RV function and improved haemodynamics.

The higher PAPi observed in the CP and 5.0 groups should not be interpreted as solely indicative of better intrinsic right ventricular function. Instead, it likely reflects the impact of adjunctive right ventricular support, which was more commonly employed in these groups.

This additional support likely contributed to improved PAPi and overall haemodynamic stabilization. Conversely, the reduction in mPAP observed in the 5.5 group highlights the device's ability to decrease LV filling pressure and provide significant unloading. These findings underscore the importance of haemodynamic variables, such as PAPi and mPAP, in identifying patients at risk for right ventricular failure after LVAD implantation. The results further suggest that mAFP preconditioning may contribute to RV function stabilization, although the observed differences in PAPi could be influenced by the differential use of adjunctive RV support.

These hemodynamic variables may help to identify patients with a high risk of developing right ventricular failure after LVAD implantation,^30^ and this study identified that a PAPi of <1.85 is a sensitive predictor of right ventricular failure after LVAD Implantation.^31^ This implies that patients undergoing mAFP preconditioning may experience stabilization of right ventricular function. The study also revealed a more pronounced increase in PAPi among individuals in the CP group compared to those with 5.0 and 5.5 L/min. However, it should be noted that these results could have been influenced by the presence of adjunctive RVAD support, which was more prevalent in the 5.0 and CP groups.

An important finding of the current study was the observed trend of liver enzyme reduction, indicating a progressive recovery of liver function potentially attributed to improved right ventricular function. An enhancement in renal and liver function postoperatively with mAFP and total body perfusion prior to LVAD implantation has been previously reported.^21^

In addition, an increase in acute renal failure was observed in patients receiving support with mAFP CP. This finding could be related to enhanced haemolysis in patients supported by low‐flow mAFPs, which could potentially predispose them to acute renal failure. Literature^32^ has already documented an increase in shear stress among patients with mAFP CP support, suggesting that this elevation in haemolysis increases the likelihood of renal failure and subsequent need for dialysis.^33^

Although there was no statistically significant difference in renal function values among the various types of mAFP in this analysis, there was evidence of lower lactate and lactate dehydrogenase levels in patients receiving higher‐flow mAFP support. There were no differences in the transfusion of blood units.

Collectively, the results suggest that a mAFP offering higher‐flow support may provide a smoother transition to the target dLVAD. However, it is essential to acknowledge that, considering the nature of the study, the statistical results could be influenced by selection bias or variations in the experience of each participating centre.

## Limitations

There are several limitations to this study. Because this is a retrospective analysis, data collection relies on accurate record keeping from others and is subject to confounding variables and selection bias. Moreover, due to the absence of a unified protocol for patient selection on mAFP, it was at the centres' discretion to identify which type of mAFP to implant. Furthermore, the prolonged duration of the study means that devices such as the Impella 5.0 or the dLVAD HeartWare are no longer available due to their relatively unfavourable outcomes.^34^ This makes the results not entirely contemporary and may affect the value of the analysis.

Additionally, it is important to emphasize that, due to the retrospective design of this study, a precise comparison of cardiogenic shock severity in each patient was not feasible. As a result, we utilized the INTERMACS classification as a standardized reference and sought to align patient groups by key descriptive characteristics. This approach aimed to reduce variability and provide a more consistent basis for comparison while acknowledging the limitations inherent to retrospective analyses.

Due to the inclusion criteria requiring patients to present with an INTERMACS 1 or 2 classification prior to mAFP implantation, these were individuals experiencing rapid deterioration on inotropic support or facing life‐threatening conditions. In such critical cases, the standard protocol across the participating centres was immediate initiation of mAFP support. This urgency in treatment protocols made it highly challenging to identify or establish a control group of patients in comparable conditions who were managed without mechanical circulatory support.

## Conclusions

These results underscore the potential advantages of utilizing higher‐flow mAFP support in patients with severe acute decompensated heart failure transitioning to durable LVAD implantation. The findings suggest improved clinical outcomes and enhanced recovery in this patient population. However, no statistically significant difference was observed between the type of mAFP used and the 30‐day mortality rate.

## Conflict of interest

Dr Oezkur and Dr Meyer reports research funding, travel funding, and speaker fees, from Abiomed. Dr Potapov reports consulting honoraria speaker honoraria, proctoring fees, and institutional grants from Abiomed, Abbot, Medtronic, and Recovery Therapeutics. Dr Treede reports advisory board honoraria from Abiomed. All other authors have reported that they have no relationships relevant to the contents of this paper to disclose. Dr A. Bernhardt, Dr Oezkur, and Dr Meyer received speaker honoraria and travel support from Abiomed. All other authors declare no competing interest.

## Funding

This research received no external funding. Open Access funding enabled and organized by Projekt DEAL.

## Data Availability

No new data were created or analysed in this study. Data sharing is not applicable to this article.
